# Robust Target Tracking with Multi-Static Sensors under Insufficient TDOA Information

**DOI:** 10.3390/s18051481

**Published:** 2018-05-08

**Authors:** Hyunhak Shin, Bonhwa Ku, Jill K. Nelson, Hanseok Ko

**Affiliations:** 1 Department of Electrical Engineering, Korea University, Seoul 02841, Korea; hhshin@ispl.korea.ac.kr (H.S.); bhku@ispl.korea.ac.kr (B.K.); 2 Department of Electrical and Computer Engineering, George Mason University, Fairfax, VA 22030, USA; jnelson@gmu.edu

**Keywords:** multi-static sonar, sonar tracking, sonar applications, MHT, tree-search algorithms

## Abstract

This paper focuses on underwater target tracking based on a multi-static sonar network composed of passive sonobuoys and an active ping. In the multi-static sonar network, the location of the target can be estimated using TDOA (Time Difference of Arrival) measurements. However, since the sensor network may obtain insufficient and inaccurate TDOA measurements due to ambient noise and other harsh underwater conditions, target tracking performance can be significantly degraded. We propose a robust target tracking algorithm designed to operate in such a scenario. First, track management with track splitting is applied to reduce performance degradation caused by insufficient measurements. Second, a target location is estimated by a fusion of multiple TDOA measurements using a Gaussian Mixture Model (GMM). In addition, the target trajectory is refined by conducting a stack-based data association method based on multiple-frames measurements in order to more accurately estimate target trajectory. The effectiveness of the proposed method is verified through simulations.

## 1. Introduction

Underwater surveillance for detecting and tracking hostile targets has often been conducted by distributed sensor networks [1,2,3]. However, new stealth technologies present challenges to detection, as the targets are increasingly becoming invisible. To illuminate invisible targets, active sensor networks are often deployed in the form of multi-static configurations wherein multiple receivers are pinged by an active source [4,5,6,7,8,9,10,11,12,13]. 

Multi-static sonar-based underwater surveillance systems can be categorized into two major types, depending on what information is used to estimate the target trajectory. The first type is a surveillance system based on line array sensors composed of multiple sensors in a linear formation, which estimates the location of the target from bearing measurements. The second type is a surveillance system composed of passive sonobouys, which determines the location of a target by calculating the time difference of arrival (TDOA) between the transmitted wave that arrives directly at the receiver and the wave that is reflected by the target [14,15,16]. TDOA measurements are also called elliptic time of arrival (TOA) of reflected signal when the travel time of the direct wave is assumed to be perfectly known [17,18,19]. Although this type of sensor is incapable of estimating bearing directly, it can obtain the range from the sensor to the target. Multiple measurements of range can be merged to estimate the location of the target.

Many conventional localization methods for multi-static sonar employ a variation of the Least Squares (LS) approach, which essentially constructs a closed-form solution from the bearing or range measurements [8,9,10]. These studies estimate the target location by fusion of measurements for 3 or more sensors, and overall tracking performance decrease significantly when insufficient measurements are available. In this case, the target localization becomes an underdetermined LS problem. In [11], to consider cases with insufficient measurements, virtual measurements are generated using Predictive Location Tracking (PLT). Based on previous target tracking results, PLT generates up to three measurements, the minimum number required for LS localization. However, due to inaccurate measurements, the virtual results risk being inaccurate, as well. In [12], target tracking with accurate localization is conducted by application of a Gaussian Mixture Model (GMM) probability model. The GMM is applied to represent and filter the uncertainty in target location caused by error in the TDOA measurements. Although this approach robustly tracks the trajectory of a target with a varying number of receivers, there is a possibility that the ghost target problem stemming from insufficient measurements may still cause performance degradation. 

In order to improve localization performance, various target tracking algorithms have been developed. Single scan approaches based on Probabilistic Data Association (PDA) have been presented to handle cluttered environment [20,21,22]. To extend PDA to multiple scan, approaches based on Multiple Hypothesis Tracking (MHT) have also been proposed [23,24,25]. However, the aforementioned approaches suffer from a heavy computational load when highly cluttered environments occur. Although improved approaches have been developed to reduce the computational load, target tracking in highly cluttered environment often fails to achieve accurate target tracking. 

To mitigate the problem caused by insufficiency and inaccuracy of TDOA information in a multi-static sonar network, this paper proposes a robust target tracking method based on a combination of track management with likelihood-based track splitting and a stack-based data association method. When insufficient measurements are observed, multiple intersections between ellipses derived from TDOA measurements are considered as possible locations of the target. We propose a track management with track splitting process to resolve the ghost target problem by composing tentative and reliable tracks. In addition, a stack-based data association method is applied to accurately estimate the trajectory of the target even in noisy environments.

The rest of this paper is organized as follows. In Section 2, we describe the model for TDOA measurements obtained from the distributed sensor network. In Section 3, we describe the proposed target tracking method based on a combination of a track management and a stack-based data association method. Section 4 provides the experimental results obtained in various simulated scenarios. Finally, conclusions are drawn in Section 5.

## 2. Problem Statement

### 2.1. Configuration of Underwater Sensor Network

**Figure 1:**
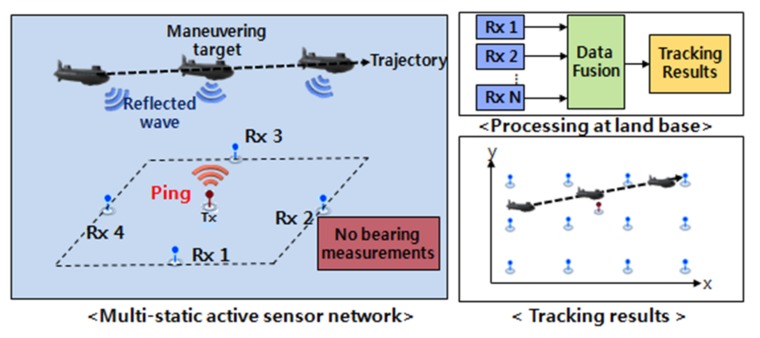
Multi-static sonar network with active pinging on sonobouy sensors in lattice formation.

As depicted in Figure 1, the multi-static sonar network system we consider is composed of one transmitter and multiple spatially distributed sonobouys. These are separated from each other and can be viewed as an extension of a bi-static sonar system with multiple receivers. It is assumed that TDOA measurements at the sonobouys are synchronized and sent to a fusion center to estimate the trajectory of the target.

### 2.2. Target Trajectory

A constant velocity model is assumed for the target. At time *t*, the state of the target is then given by,

(1) $$x_{t}=Fx_{t-1}+w_{t}$$

where **x***_t_* = [**p***_t_*, **v***_t_*] is the target state (position and velocity), *w_t_* is independent and identically distributed (i.i.d). zero mean Gaussian noise with covariance $\Sigma_{w}$ at time *t*. The state transition matrix, **F**, is given by

(2) $$F=\left[\begin{array}{cccc} 1 & 0 & T & 0\\ 0 & 1 & 0 & T\\ 0 & 0 & 1 & 0\\ 0 & 0 & 0 & 1 \end{array}\right]$$

where *T* is the time interval at which measurements are obtained.

### 2.3. TDOA Measurements

At the *i*-th receiver, the TDOA measurement, $\tau_{t,i}$ , between the direct wave and a target-reflected wave at time *t* can be represented by the following equation:

(3) $$\tau_{t,i}=\frac{\left\|d_{t,i}\right\|+\left\|e_{t,i}\right\|-\left\|b_{i}\right\|}{c}+n_{t,i}$$

where **d***_t_*_,*i*_, **e***_t,i_*, **b***_i_*, *n_t,i_* and *c* are the displacement of the incident wave, the displacement of the reflected wave, the displacement of the direct wave, the noise of TDOA measurement and the velocity of the sound wave, respectively. 

**Figure 2:**
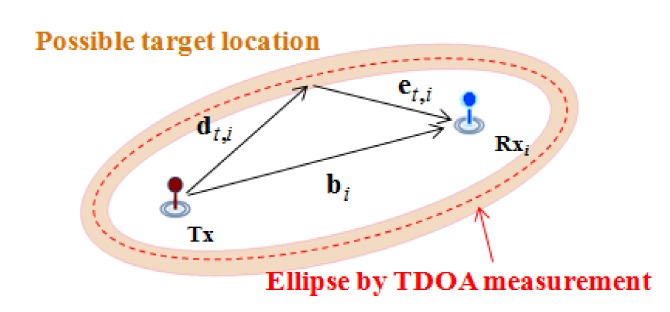
Estimated target location using TDOA measurement.

By assuming that *c* is constant, the sum of distances of the incident and reflected wave can be calculated from the acquired TDOA measurement. Therefore, as shown in Figure 2, the estimated position of the target using the TDOA measurement can be represented as an ellipse trace with uncertainty due to measurement error depicted by the shaded region. 

### 2.4. GMM Representation of TDOA Measurement

**Figure 3:**
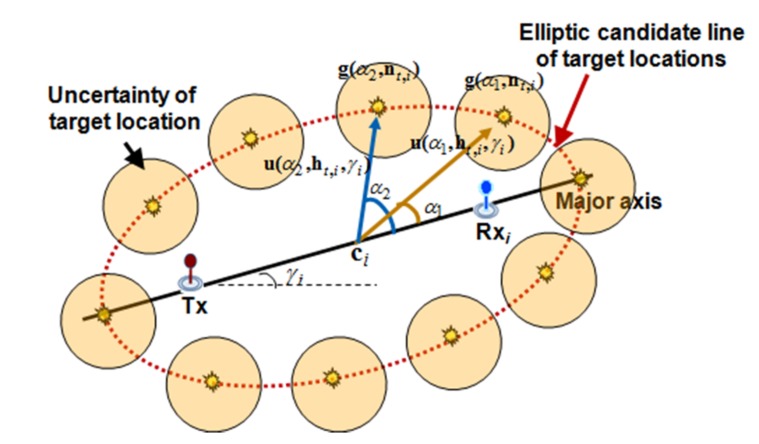
GMM Representation for uncertainty of target location using TDOA measurement.

The uncertainty around the estimated ellipse can be represented using GMM as shown in Figure 3, and the discrete locations on the estimated ellipse can be represented as a parametric form of direction and distance as shown in Equation (4) [12,13].

(4) $$g(\alpha_{k},a_{t,i})=c_{i}+u(\alpha_{k},h_{t,i},\gamma_{i})$$

where *α_k_* is the direction between the center of the ellipse and the *k*-th discrete location, and the **a***_t_*_,*i*_ is the vector, [**c***_i_*, **h***_t_*_,*i*_, *γ*_i_]^T^, which represents the geometric information for the TDOA ellipse of the *i*-th receiver at time *t* (see Figure 3). Let **c***_i_*, **h***_t_*_,*i*_, *γ*_i_ denote the coordinate location of the center, distance of the axis, and angle of the ellipse, respectively.

(5) $$u(\alpha_{k},h_{t,i},\gamma_{i})=\;{\left[\begin{array}{c} h_{t,i}^{1}\cos\alpha_{k}\\ h_{t,i}^{2}\sin\alpha_{k} \end{array}\right]}^{T}\left[\begin{array}{cc} \cos\gamma_{i} & \sin\gamma_{i}\\ -\sin\gamma_{i} & \cos\gamma_{i} \end{array}\right]$$

Let the **u**(*α_k_*, **h***_t_*_,*i*_, *γ*_i_) be the vector that represents the displacement between the center coordinate and *k*-th discrete location on the *i*-th estimated ellipse and **h**^1^*_t_*_,*i*_, **h**^2^*_t_*_,*i*_ denote the distance of the major and minor axis, respectively, of the *i*-th ellipse at time *t*.

Using GMM, the likelihood of target position **p**_t_ is expressed as the weighted sum of the component densities composed of discrete locations **g**(*α_k_*, **a***_t_*_,*i*_) on the ellipse covariance **R***_k_*_,*i*_, i.e.,

(6) $$P(p_{t}|\tau_{t,i})=\sum_{k=1}^{G}w_{k,i}N\left(p_{t};g(\alpha_{k},a_{t,i}),\;R_{k,i}\right)$$

where *N*(**p**_t_; **g**(*α_k_*, **a***_t_*_,*i*_), **R***_k_*_,*i*_) denotes the Gaussian pdf of variable **p**_t_ with mean **g**(*α_k_*, **a***_t_*_,*i*_) and variance **R***_k_*_,*i*_ and *w_k_*,*_i_* is the weight of the *k*-th component of the Gaussian mixture. *G* denotes the fixed number of Gaussian mixture components. In this paper, the value of *G* was chosen experimentally. A detailed description of how the covariance and weight for each Gaussian mixture component [12] is presented as follows. To approximate the likelihood, one sigma range of the TDOA measurement is considered. The segmentation of each component according to one sigma range is defined as follows,

(7) $$\left[\begin{array}{c} q_{1}\\ q_{2}\\ q_{3}\\ q_{4} \end{array}\right]=\left[\begin{array}{c} c_{i}+u(\alpha_{k},[h_{t,i}^{1}+\delta_{i}^{1},h_{t,i}^{2}+\delta_{i}^{2}],\gamma_{i})\\ c_{i}+u(\alpha_{k+1},[h_{t,i}^{1}+\delta_{i}^{1},h_{t,i}^{2}+\delta_{i}^{2}],\gamma_{i})\\ c_{i}+u(\alpha_{k},[h_{t,i}^{1}-\delta_{i}^{1},h_{t,i}^{2}-\delta_{i}^{2}],\gamma_{i})\\ c_{i}+u(\alpha_{k+1},[h_{t,i}^{1}-\delta_{i}^{1},h_{t,i}^{2}-\delta_{i}^{2}],\gamma_{i}) \end{array}\right]$$

where $\delta_{i}^{1}$ and $\delta_{i}^{2}$ are the increments of the major and minor axis according to the one sigma range of TDOA measurement at *i*-th sensor. Then length *D_c_* and unit vector are respectively defined by the following equations,

(8) $$D_{c}=\left\|\Delta q_{c}\right\|/2,\;\;\;\;\;\Delta q_{c}=(q_{1}+q_{3}-q_{2}-q_{4})/2$$

(9) $$i\left(\alpha_{c}\right)=\frac{\Delta q_{c}}{\left\|\Delta q_{c}\right\|}$$

The length *D*_0_ of the other semi axis is given by following equation,

(10) $$D_{0}=i{\left(\alpha_{c}+\pi /2\right)}^{T}(\frac{q_{1}+q_{2}-q_{3}-q_{4}}{4})$$

Then covariance matrix $R_{k,i}$ is defined as,

(11) Rk,i=[i(α)  i(α+π/2)]T[Dc200D02][i(α)  i(α+π/2)] 

Finally, the weight for each Gaussian mixture is proportional to the area of the covariance matrix.

(12) $$w_{k,i}=\sqrt{\left|R_{k,i}\right|}\;$$

## 3. Proposed Algorithm

### 3.1. Fusion of TDOA Measurement Uncertainty

Since the underwater sensor network we consider is composed of multiple receivers, it is necessary to expand the likelihood function of a single receiver in Equation (6) to multiple TDOA measurements. A method that fuses TDOA measurements represented by GMMs was proposed in [12], where the joint likelihood was defined as,

(13) $$P(p_{t}|\tau_{t,1},\tau_{t,2},\ldots ,\tau_{t,N_{t}})=\sum_{k=1}^{G}w\prime_{k}N(p_{t};g\prime_{t,k},R\prime_{t,k})$$

The approximated mean, covariance and weight of the fused mixture component is defined by the multiplication of mixture pairs for each GMM,

(14) $${\left(R\prime_{t,k}\right)}^{-1}=\sum_{i=1}^{N_{t}}{\left(R_{B_{t,i}^{k},i}\right)}^{-1}$$

(15) $$g\prime_{t,k}=R\prime_{t,k}\sum_{i=1}^{N_{t}}{\left(R_{B_{t,i}^{k},i}\right)}^{-1}g(\alpha_{B_{t,i}^{k}},a_{t,i})$$

(16) $${\hat{w}}_{k}\approx \prod_{i=1}^{N_{t}}w_{B_{t,i}^{k},i}\;\;\;,\;\;\;\;w\prime_{k}=\frac{\;{\hat{w}}_{k}}{\sum_{k=1}^{G}{\hat{w}}_{k}}$$

where *N_t_* is the total number of TDOA observations, $B_{t,i}^{k}$ is the index for the *k*-th mixture pair of *i*-th GMM determined by measurement selection strategies of target tracking sensor suite depicted in [26] at time *t*.

However, considering all mixture components incurs unnecessary computation. In addition, because some mixtures are unrelated to the target position, using all mixture components may degrade localization performance. In order to reduce computation and increase localization performance, a fusion method using only the mixture components near the intersections is proposed. The fused likelihood can be described by the following equation:

(17) $$P(p_{t}|\tau_{t,1},\tau_{t,2},\ldots ,\tau_{t,N_{t}})\;\approx \;\sum_{n=1}^{M_{t}}{\bar{w}}_{n}N(p_{t};{\bar{g}}_{t,n},{\bar{R}}_{t,n})$$

where *M_t_* is the number of fused mixture components and ${\bar{w}}_{n}$ , ${\bar{g}}_{t,n}$ , ${\bar{R}}_{t,n}$ are the approximated weight, mean and covariance, respectively. The parameters of each fused mixture component are calculated by the multiplication of selected mixture components near the intersections. The detailed process for selecting mixture components based on intersections is described in the next paragraph.

**Figure 4:**
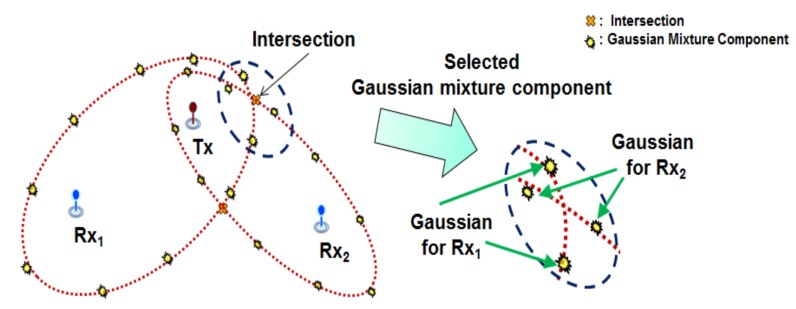
Mixtures selection for two TDOA measurements.

When two TDOA measurements are obtained from the multi-static sensor networks, intersections of the two ellipses are estimated (see Appendix A). For example, the number of fused mixture components is two and the four neighboring mixture components from the each intersection are selected as in Figure 4.

**Figure 5:**
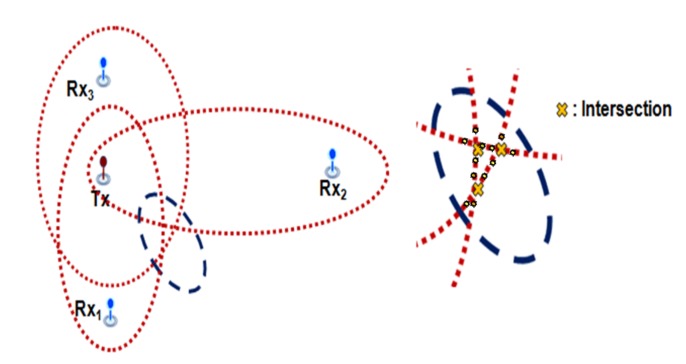
Mixture selection for three TDOA measurements.

If we obtain three or more TDOA measurements, intersections of three or more ellipses may occur, as shown in Figure 5. In this situation, multiple neighboring mixture components from these intersections are selected for one fused mixture. In order to obtain the locations of these intersections, we use the Euclidean distance between the intersections as given in,

(18) $$E_{t,q}=\{\;I_{t,r}:{\left\|I_{t,q}-I_{t,r}\right\|}_{}<\varepsilon ,\;q\neq r\;\}$$

where **I***_t_*,*_q_* and I*_t_*,*_r_* are the locations of intersections at time *t*, and *ε* is tolerance error. The elements of the set **E***_t_*,*_q_* are selected as the mixture components for computing the fused likelihood. For intersections of three ellipses as shown in Figure 5, for example, we can select twelve neighboring mixtures.

### 3.2. Track Management for Insufficient Information

**Figure 6:**
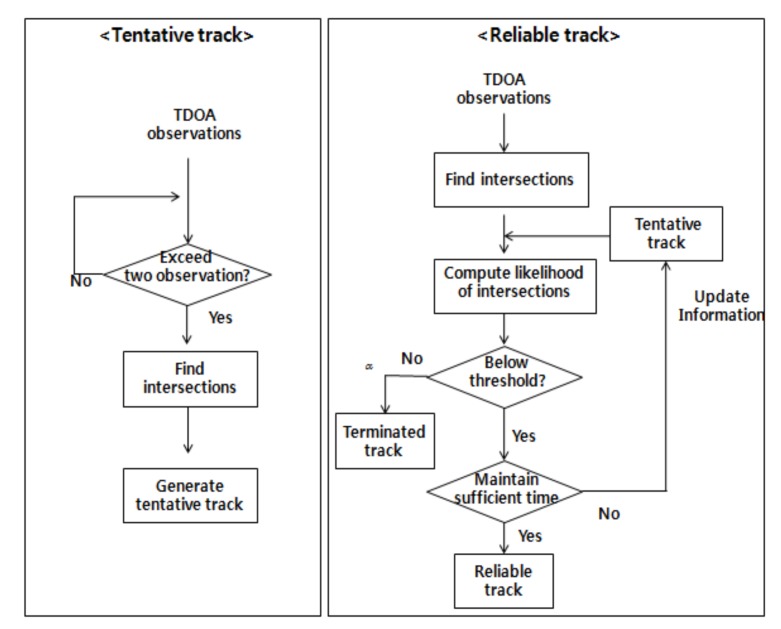
Flow diagram of proposed track management.

When monitoring a large surveillance region, ambiguity in target location occurs due to the insufficient measurement information produced by a limited number of sensors. Because all intersections of TDOA ellipses have the same likelihood value, it is difficult to determine the intersection that indicates the true position of the target. If this ambiguity is not resolved, tracking performance will likely be degraded due to the presence of ghost targets. In this paper, track management with track splitting based on the likelihood function is proposed in order to suppress the influence of the ambiguity caused by ghost targets, as discussed in our previous work [27]. In order to resolve the ambiguity, it is necessary to establish a reliable track for the target as described in Figure 6. First, tentative tracks are initiated at the ellipse intersections when two or more TDOA measurements are received from the sensor network. Then, the status of each tentative track can be transitioned into either a confirmed track or a terminated track through the process of track management in subsequent scans.

Given the *l*-th sequence of intersections up to time *t*, the likelihood that a particular track is a true target track can be written as,

(19) $$\Lambda (\pi_{t}^{l})=p(I_{1}^{l},I_{2}^{l},\ldots ,I_{t}^{l}|\pi_{t}^{l})$$

where $\pi_{t}^{l}$ is an event and $I_{t}^{l}$ is the intersection location of the *l*-th sequence at time *t*. Under independent linear-Gaussian assumption, the likelihood function can be approximated:

(20) $$\Lambda (\pi_{t}^{l})=\prod_{j=1}^{t}p(I_{j}^{l}|I_{1}^{l},\ldots .,I_{j-1}^{l},\pi_{t}^{l})=\prod_{j=1}^{t}N(I_{j}^{l};H{\hat{I}}_{j|j-1}^{l},S_{j}^{l})$$

where $H=[\begin{array}{cc} 1 & 0 \end{array}]$ represents the relation between state and locations. $H{\hat{I}}_{j|j-1}^{l}$ and $S_{j}^{l}$ are the predicted location and innovation covariance, respectively, at time *j*.

(21) $${\hat{I}}_{j|j-1}^{l}=F{\hat{I}}_{j-1|j-1}^{l}$$

(22) $$S_{j}^{l}=H^{T}C_{j|j-1}^{l}H+R_{j}^{l}$$

where $C_{j-1|j-1}^{l}$ is the covariance of the predicted state and $R_{j}^{l}$ is the covariance of the fused mixture component located near the intersection. The mixture component located near the intersection of three or more ellipses has low covariance relative to the component at the intersection of two ellipses due to the fusion of a larger number of selected mixture components.

Following from Equation (20), the negative log likelihood of the track can be approximated recursively as follows:

(23) $$\lambda (\pi_{t}^{l})=\lambda (\pi_{t-1}^{l})+{\left(I_{t}^{l}-{\hat{I}}_{t|t-1}^{l}\right)}^{T}{\left(S_{t}\right)}^{-1}(I_{t}^{l}-{\hat{I}}_{t|t-1}^{l})$$

If the track negative log likelihood is below a threshold during a certain period of time, the tentative track is transitioned into the reliable track. Otherwise, the tentative track is terminated. This threshold follows from the chi-square table where the tail probability is typically determined as 0.01 [28]. 

When insufficient measurements are obtained in the update process, intersections representing false (ghost) targets can be eliminated via a gating process:

(24) $${\left(d_{t,n}\right)}^{2}={\left(I_{t,n}-{\hat{I}}_{t|t-1}\right)}^{T}S_{t}{}^{-1}(I_{t,n}-{\hat{I}}_{t|t-1})$$

where **I***_t_*_,*n*_ is the location of the *n*-th intersection, and *d_t_*_,*n*_ is the Mahalanobis distance between the predicted location derived from the reliable track and intersection locations at time *t*. If the Mahalanobis distance exceeds a certain threshold, we consider the intersection to be a false location. Like the reliable track process, the threshold is obtained from the Chi-square table [28].

### 3.3. Track Estimation Using Stack-Based Data Association

In a dense and noisy environment, the target localization performance can often be degraded abruptly. In this situation, in order to improve the localization performance, a batch tracking method based on measurements from multiple scans, rather than single scan, is considered. 

**Figure 7:**
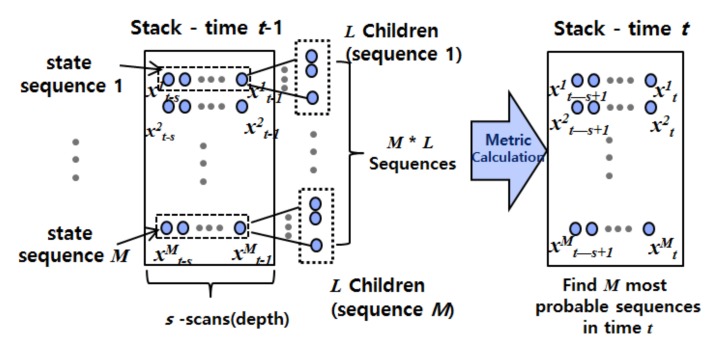
Schematic for stack-based data association.

We propose a tree search-based method for tracking based on a sequence of measurements, or scans. Specifically, we apply a variant of the stack algorithm [29,30], a best-first approach that extends the most likely path (or *L* paths) at each iteration of the algorithm. The proposed stack-based data association method uses a tree structure and builds a list (or stack) of possible state sequences containing states derived from acceptable target locations. The recursive steps of stack-based data association are as shown in Figure 7. At each time, all target state sequences in the stack are extended to *L* possible children representing *L* possible current states. The trajectory of a target is then estimated by a weighted sum of only the *M* most likely target state sequences of *M* × *L* sequences. Here, the weight value of each target state sequence is directly proportional to its posterior probability calculated from measurements, which will be described later.

In the proposed method, we find the trajectory through a set of candidate locations in a target existence region established from the approximated likelihood function of sequence of intersections from track management. The center location and validation limit of the target existence region apply the approximated mean and covariance, respectively. At time *t*, the measurements of the target are represented as the candidate locations sampled from the target existence region,

(25) $$Z_{t,s}=\{Z_{t-s+1,},\ldots ,Z_{t}\},\;\;Z_{t}=\{z_{t}^{1},z_{t}^{2},\ldots ,z_{t}^{v}\}$$

where **Z***_t_*,*_s_* denotes the set of measurements from consecutive scans, *t* is the current time, and *s* is the scan depth. $z_{t}^{v}$ is sampled uniformly in the target existence region with approximated likelihood, and *v* is the number of candidate locations. It can be assumed that the sampled measurements in the target existence region have originated from the target or from clutter. Therefore, the association event of the *i*-th measurement at time *t* is defined as

(26) $$\theta_{t}^{i}=\langle \begin{array}{c} \{\;z_{t}^{i}\;\mathrm{is}\;\mathrm{the}\;\;\mathrm{measurement}\;\;\mathrm{associated}\;\;\mathrm{with}\;\mathrm{target}\;\}\;\;\;\;\;\;\;i=1,2,\ldots ,v\\ \{\;\mathrm{None}\;\mathrm{of}\;\mathrm{measreuements}\;\;\mathrm{is}\;\;\mathrm{associated}\;\;\mathrm{with}\;\mathrm{target}\;\}\;\;\;\;\;\;\;\;\;\;\;\;i=0 \end{array}$$

The problem of target trajectory estimation using a candidate measurement sequences is based on approximation of a posteriori probability density function of a target state sequence. A brute force approach to this problem is to establish a tree structure with the measurements from the batch data and perform an exhaustive search of the paths through the tree to select the most likely sequence of candidates. However, the number of possible sequences increases exponentially in highly cluttered environments. Although many approaches, including pruning and merging, have been applied, these approaches have not provided sufficient performance in extreme conditions. The proposed stack-based data association overcomes this problem by searching for a sequence of target states instead of a sequence of measurements.

In order to establish the state sequences in the stack, a discretization process that samples possible child states is required. In the discretization process, *L* possible child states of each sequence in the stack are sampled uniformly; only the validation region (rather than the full state space) is considered in order to reduce estimation complexity. At time *t*, the predicted state $x_{t|t-1}^{k}$ and the innovation covariance of the target state sequences are calculated as like as in Equations (21) and (22). Here, the validation regions for target state sequences are determined by using the predicted mean and its covariance as in Equation (24).

Since the weight of the *k*-th sequence of target states in the stack is proportional to the posterior probability conditioned on *s* measurements from consecutive scans, we can write this posterior probability using Bayes’ theorem as,

(27) $$P(X_{t,s}^{k}|Z_{t,s})=\frac{P(Z_{t,s}|X_{t,s}^{k})P(X_{t,s}^{k})}{P(Z_{t,s})}$$

where $X_{t,s}^{k}=\{x_{t-s+1}^{k},x_{t-s+2}^{k},\cdots ,x_{t}^{k}\}$ , and *P*(**Z***_t_*,*_s_*) is a normalization term that is assumed to be constant across time. *P*(**Z***_t_*,*_s_*|**X**^k^*_t_*,*_s_*) is the cumulative likelihood term of consecutive measurements conditioned on target state sequences. *P*(**X**^k^*_t_*,*_s_*) is the prior on the target state sequence, all of which are assumed to be equally likely. The weight $P(X_{t,s}^{k}|Z_{t,s})$ of the *k*-th target state sequence is calculated as,

(28) $$\begin{array}{l} P(X_{t,s}^{k}|Z_{t,s})\propto P(Z_{t,s}|X_{t,s}^{k})P(X_{t,s}^{k})\\ \;\;\;\;\;\;\;\;\;\;\;\;\;\;\;\;\;\;\;\;\;=\prod_{j=t-s+2}^{t}p(Z_{j}|x_{j}^{k})\times \prod_{m=t-s+1}^{t-1}q(x_{m+1}^{k}|x_{m}^{k}) \end{array}$$

where $q(x_{m+1}^{k}|x_{m}^{k})$ is the state transition probability which follows a Gaussian distribution with mean $Fx_{m}^{k}$ and covariance $\Sigma_{w}$ .

(29) $$q(x_{m+1}^{k}|x_{m}^{k})=N(x_{m+1}^{k};Fx_{m}^{k},\Sigma_{w})$$

The cumulative conditional likelihood *p*(**Z***_j_*|**x***^k^_j_*) is calculated by summing over all association events as follows:

(30) $$p(Z_{j}|x_{j}^{k})=\sum_{i=0}^{m_{j}}p(\theta_{j}^{i}|x_{j}^{k})\prod_{r=1}^{m_{j}}p(z_{j}^{r}|x_{j}^{k},\theta_{j}^{i})$$

where *m*_j_ is the number of validated measurement at time *j.* Note that the second line follows from the independence Gaussian assumption. The observation probability is given by

(31) $$p(z_{j}^{r}|x_{j}^{k},\theta_{j}^{i})=\{\begin{array}{c} N(z_{j}^{r};Hx_{j}^{k},\Sigma_{v})\;\;\;\;\;\;\;\;\;\;\;\;\;\;\;\;\;\;for\;\;i=r\\ \frac{1}{V}\;\;\;\;\;\;\;\;\;\;\;\;\;\;\;\;\;\;\;\;\;\;\;\;\;\;\;\;\;\;\;for\;\;i\neq r \end{array}$$

where $\Sigma_{v}$ is the covariance of the observation matrix **H**. When index *i* is equal to index *r*, the probability represents the *r*-th valid measurement which was generated by the state **x***^k^_j_*. Therefore, it is calculated from the distribution of the observation model. Otherwise, the clutter measurements are assumed to be uniformly distributed in the validation region with volume *V*. The prior probability of each association event and is given by

(32) $$p(\theta_{j}^{i}|x_{j}^{k})=\{\begin{array}{c} P_{D}\;\mu_{F}(m_{j}-1)\;\;\;\;\;\;\;\;\;\;\;\;\;\;\;\;\;\;\;\;\;for\;\;i\neq 0\\ (1-P_{D})\mu_{F}(m_{j})\;\;\;\;\;\;\;\;\;\;\;\;\;\;\;\;\;for\;\;i=0 \end{array}$$

where *P_D_* is the probability of detecting the target. Let $\mu_{F}(m_{j})$ denote the probability mass function of *m*_j_ false measurements in the validation region modeled by a Poisson distribution [28],

(33) $$\mu_{F}(m_{j})\;=e^{-\lambda V}\frac{{\left(\lambda V\right)}^{m_{j}}}{m_{j}!}$$

where *λ* is the average number of clutter measurements per unit volume.

## 4. Performance Evaluation

### 4.1. Experimental Environment

**Figure 8:**
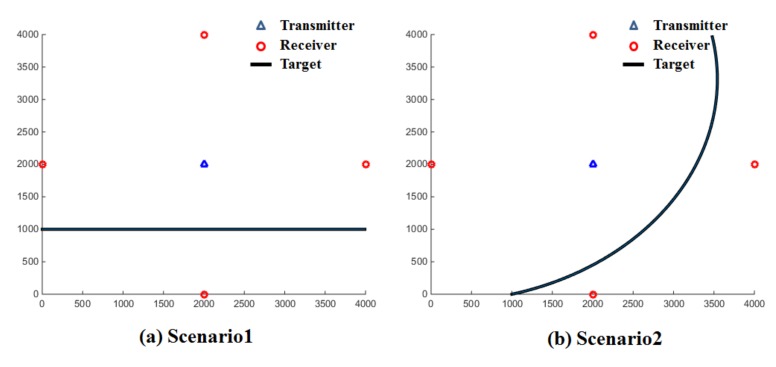
Simulation target scenarios.

As displayed in Figure 8, the scenarios we consider include a transmitter at the center and four sonobuoy receivers equally spaced around the boundary of the 4 × 4 km surveillance region. The first scenario assumes that a target moves in a straight line from the lower-left side of the surveillance region to the lower-right side at a speed of 10 knots. The second scenario considers a target moving in a curved line from the lower-left side to the upper-right side of the surveillance region, again at 10 knots. 

The coverage of each of the deployed receivers was determined under the active sonar equation as follows [31]:

(34) $$SNR=SL-TL_{1}-TL_{2}-NL+TS\geq DT$$

where SNR indicates signal-to-noise ratio. Details of the parameters in Equation (34) are summarized in Table 1.

**Figure 9:**
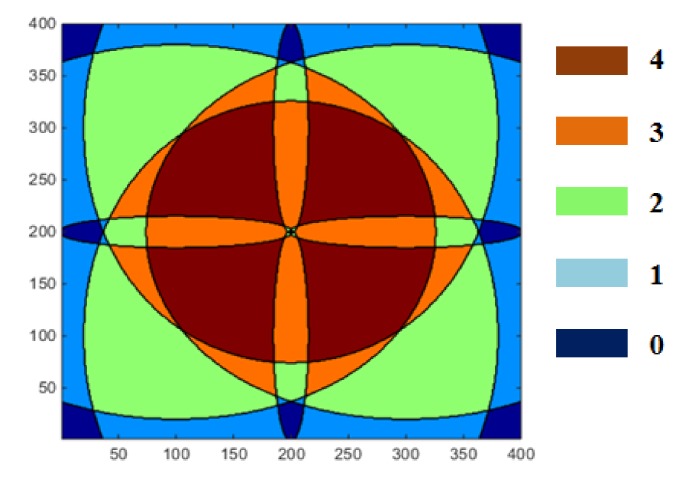
Sensor detection coverage for simulation.

If Target Strength (*TS*), the parameter related to the aspect angle and direction of the target, is assumed to be constant, the target detection range of the sensor pair is determined by Transmission Loss (*TL*), which refers to the distance between the target and the sensor pair. The coverage and overlapping of the sensor network is displayed in Figure 9. The color stands for the number of sensors overlapped sensor coverage. Brown, orange, green, and sky blue color indicates the coverage of four, three, two and one receivers, respectively.

We evaluated the effectiveness of the proposed algorithms in terms of the average RMSE (Root Mean Squared Error) in target location estimation. Here, the average RMSE is defined by

(35) $$\mathrm{average}\;\mathrm{RMSE}=\frac{1}{A}\sum_{t=1}^{A}\sqrt{\frac{1}{N_{s}}\sum_{i=1}^{N_{s}}{\left\|p_{t}-{\hat{p}}_{t,i}\right\|}_{2}^{2}}$$

where *A* is the total number of scans, *N*_S_ is the total number of Monte Carlo runs, and ${\hat{p}}_{t,i}$ is the estimated location of the target at time *t* in the *i*-th iteration. Simulation results are conducted with MATLAB using a Monte-Carlo method with 1000 iterations.

### 4.2. Discussion of Experimental Reults

**Figure 10:**
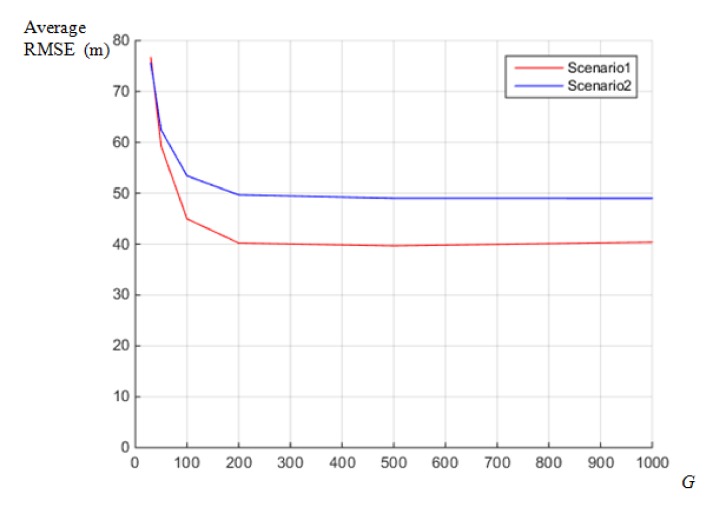
Performance assessments on value of *G*.

Figure 10 shows the average RMSE of localization in terms of *G* in Equation (6), which refers to the number of Gaussian mixture components. In the experiment, we assumed identical measurement error for the four receivers and standard deviations of the TDOA measurement error of 0.03 s. As shown in Figure 10, the average RMSE decreases as the number of mixture components increases. However, for values of *G* exceeding 200, the localization performance has converged in both scenarios. Therefore, we set the value of *G* to 200 in all experiments below.

Table 2 shows the tracking performance in terms of managing the reliable track based on the track splitting algorithm operating on insufficient TDOA measurements. As shown in Table 2, the GMM-based fusion method is incapable of determining the accurate location of the target when ghost targets occur. However, by managing the reliable track, it is shown that the proposed method robustly tracks the location of the target by suppressing the false intersection.

The tracking procedure is conducted by data association of the multiple candidates generated in the fused likelihood at each scan. In the following experiments, the proposed method based on stack management is compared with localization by GMM [12] with track management, the Probability Data Association (PDA) algorithm, which is a single scan processing method [20], and the history-based Multiple Hypothesis Tracking (MHT) algorithm, which is a batch data processing method [23]. Details of the basic parameters of tracking algorithms are summarized in Table 3.

The proposed method conducts target tracking from the reduced set of candidate locations in the target existence region generated by TDOA measurement fusion. In order to evaluate performance as a function of the size of the target existence region, we constrain the sizes of the regions according to the thresholds of Mahalanobis distance that were calculated from *g*-sigma values with 2 degrees of freedom [28]. In Table 4 and Table 5, as the *g*-sigma value increases, the target existence region is also enlarged. To consider various noisy environments, the standard deviations of the TDOA measurement error referring to the relatively low and high noise environment were 0.03 s and 0.07 s, respectively. The number of candidate locations in the target existence region was also set to 17. According to results, in the case of a relatively low-noise environment, it is observed that the overall average RMSE increases as the size of the target existence region increases. In addition, when the value of *g*-sigma is 3, the average RMSE of both the PDA, MHT method and the proposed method rapidly increase. However, in case of a relatively high-noise environment, it is observed that increasing the size of the target existence region is appropriate for handling the high-noise case. Especially when the *g*-sigma value is higher than 2, it is shown that the performance of both methods is better than the 1-sigma case. Therefore, it is possible to say that setting an appropriate distance threshold of the target existence is important, and the proper *g*-sigma value for the simulation considered is 2.

For performance assessment of the proposed method, varying the number of candidate locations was considered, and results are shown in Table 6 and Table 7. The *g*-sigma value for determining the target existence region was again set to 2. As shown in Table 6 and Table 7, results indicate that the proposed method is as effective as MHT in both scenarios. The performance of the proposed method in the relatively low-noise environment is comparable to MHT, while in the case of a relatively high-noise environment, the performance of the proposed method is significantly superior to the MHT method, particularly for scenario 2. Though a constant velocity model was assumed in this simulation, the proposed method could robustly track the curved line maneuvering target by managing the multiple candidates of sequences of the target state. In addition, it is shown that increasing the number of candidates in the target existence region could improve tracking performance. However, as the number of candidates increases, it is also observed that the performance improvement gradually dwindles.

Table 8 indicates the computational time required for each method for one scan of data. In the case of 5 candidate locations, the proposed method requires a similar amount of computation time to MHT. However, as the number of candidate locations increases, it is observed that the difference in computation time between MHT and the proposed method rapidly increases. In dense candidate locations, the proposed method is vastly superior to MHT in the sense of computational load due to its concept of maintaining a certain number of highly probable target states.

## 5. Conclusions

In scenarios in which the surveillance region of the multi-static sensor network is extensive and only a small number of receivers are deployed in noisy underwater environments, the coverage of the sensor network is reduced, resulting in insufficient and inaccurate data for target tracking. In this paper, a method that robustly tracks the trajectory of an underwater target using insufficient and inaccurate information collected from a sensor network is proposed.

The proposed method seeks to solve this problem by following two procedures. First, track management with a track splitting algorithm is proposed to eliminate the influence of the ghost target caused by insufficient TDOA measurements. Second, an efficient method for fusing the uncertainty of multiple TDOA measurements (represented by GMM) based on intersections is proposed. In addition, using a stack-based data association method with candidates in a fused likelihood of multiple scans, inaccurate target location is refined. It is observed via simulations that the proposed tracking method is capable of robustly tracking the target even in high-noise conditions. In addition, in comparison with MHT, the target tracking results of the proposed method showed improvements in both accuracy and computational complexity.

## Figures and Tables

**Table 1:** Details of parameters for sonar equation.

Parameter	Value
Signal Level (*SL*)	185 dB
Detection Threshold (*DT*)	10 dB
Noise Level (*NL*)	70 dB
Target Strength (*TS*)	10 dB
Sound Velocity (*c*)	1500 m/s

**Table 2:** Average RMSE according to management of reliable track.

Average RMSE (m)	GMM	GMM + Track Management
Scenario 1	77.24	31.52
Scenario 2	95.51	40.74
Average	86.37	36.13

**Table 3:** Common parameters for tracking in experiments.

Parameter	Value
Covariance matrix of state transition	diag(5,5,1,1)
Covariance matrix of observation	diag(10,10)
Detection rate (*P_D_*)	0.9
Scan Depth (*s*)	5
Possible children (*L*)	9
Maximum number of stack entries (*M*)	128

**Table 4:** Average RMSE for varying the size of target existence region for scenario 1.

Measurement Error (m)	*g*-Sigma	Tracking Method			
		GMM [12]	PDA [20]	MHT [23]	Proposed
Relatively low	1	31.52	24.52	20.07	20.15
	2		25.77	21.12	20.37
	3		28.13	24.25	22.15
Relatively high	1	61.42	52.76	46.22	42.32
	2		50.65	42.18	39.28
	3		50.81	42.77	39.15

**Table 5:** Average RMSE for varying the size of target existence region for scenario 2.

Measurement Error (m)	*g*-Sigma	Tracking Method			
		GMM[12]	PDA [20]	MHT [23]	Proposed
Relatively low	1	40.74	35.11	31.52	31.22
	2		36.13	31.92	31.46
	3		38.71	34.01	33.16
Relatively high	1	73.45	65.31	61.21	55.92
	2		64.06	58.01	51.37
	3		64.34	57.52	51.12

**Table 6:** Average RMSE for varying the number of candidate location for scenario 1.

Measurement Error	Candidate Number	Tracking Method			
		GMM [12]	PDA [20]	MHT [23]	Proposed
Relatively low	5	31.52	26.51	22.51	22.12
	9		26.33	21.72	21.05
	13		26.11	21.34	20.52
	17		25.77	21.12	20.37
Relatively high	5	61.42	52.72	45.72	45.22
	9		51.87	43.28	42.27
	13		51.11	42.61	40.51
	17		50.65	42.18	39.28

**Table 7:** Average RMSE for varying the number of candidate location for scenario 2.

Measurement Error	Candidate Number	Tracking Method			
		GMM [12]	PDA [20]	MHT [23]	Proposed
Relatively low	5	40.74	37.21	33.52	33.74
	9		37.11	32.25	32.55
	13		36.67	32.04	31.82
	17		36.13	31.92	31.46
Relatively high	5	73.45	65.55	63.21	58.79
	9		64.12	59.21	53.12
	13		64.25	58.44	51.72
	17		64.06	58.01	51.37

**Table 8:** Comparison of computational time for one scan data.

Candidate Number	Processing Time (s)	
	MHT [23]	Proposed Method
5	0.54	0.58
9	2.12	1.11
13	4.25	1.45
17	7.12	1.72

## Appendix A

In order to estimate the location of the target from the TDOA measurements, it is necessary to compute the location of intersections between the TDOA ellipses. The locations of intersections are determined by sequentially considering all possible pairs of TDOA measurements. The *i*-th TDOA ellipse can be written as Equation (A1)

(A1)
$$\frac{{\left(x\cos\gamma_{i}-y\sin\gamma_{i}-c_{i}^{1}\right)}^{2}}{h_{t,i}^{1}}+\frac{{\left(x\sin\gamma_{i}+y\cos\gamma_{i}-c_{i}^{2}\right)}^{2}}{h_{t,i}^{2}}=1$$

where *x*, *y* denote the coordinate variables of TDOA ellipses. Then two ellipses, the *i*-th and *j*-th ellipse, can be arranged as in the following quadratic form.

(A2)
$$\begin{array}{l} Q_{i}(x,y)=s_{0}+s_{1}x+s_{2}y+s_{3}x^{2}+s_{4}xy+s_{5}y^{2}=0\\ Q_{j}(x,y)=l_{0}+l_{1}x+l_{2}y+l_{3}x^{2}+l_{4}xy+l_{5}y^{2}=0 \end{array}$$

The quadratic equations can be viewed as quadratic polynomials in *x* with coefficients that depend on *y*.

(A3)
$$\begin{array}{l} Q_{i}(x,y)=(s_{0}+s_{2}y+s_{5}y^{2})+(s_{1}+s_{4}y)x+s_{3}x^{2}=\delta_{0}+\delta_{1}x+\delta_{2}x^{2}=0\\ Q_{j}(x,y)=(l_{0}+l_{2}y+l_{5}y^{2})+(l_{1}+l_{4}y)x+l_{3}x^{2}=\omega_{0}+\omega_{1}x+\omega_{2}x^{2}=0 \end{array}$$

The common root of the two quadratic polynomials in Equation (A3) satisfies the following equations:

(A4)
$$\delta_{2}Q_{j}(x,y)-\omega_{2}Q_{i}(x,y)=(\delta_{2}\omega_{1}-\delta_{1}\omega_{2})x+(\delta_{2}\omega_{0}-\delta_{0}\omega_{2})=0$$

(A5)
$$\delta_{1}Q_{j}(x,y)-\omega_{1}Q_{i}(x,y)=(\delta_{1}\omega_{2}-\delta_{2}\omega_{1})x^{2}+(\delta_{1}\omega_{0}-\delta_{0}\omega_{1})=0$$

Then Equation (A6) is constructed by eliminating the variable *x* in Equations (A4) and (A5),

(A6)
$$(\delta_{2}\omega_{1}-\delta_{1}\omega_{2})(\delta_{1}\omega_{0}-\delta_{0}\omega_{1})-{\left(\delta_{2}\omega_{0}-\delta_{0}\omega_{2}\right)}^{2}=0$$

The quartic equation for variable *y* is constructed by substituting the variable in Equation (A3) into Equation (A6). The real roots of this quartic equation represent the *y* locations of intersections. Finally, the corresponding *x* location is calculated in Equation (A7) by using the value *y*.

(A7)
$$x=\frac{\delta_{2}\omega_{0}-\delta_{0}\omega_{2}}{\delta_{1}\omega_{2}-\delta_{2}\omega_{1}}$$
